# *Child-Sum EATree-LSTMs*: enhanced attentive *Child-Sum Tree-LSTMs* for biomedical event extraction

**DOI:** 10.1186/s12859-023-05336-7

**Published:** 2023-06-15

**Authors:** Lei Wang, Han Cao, Liu Yuan, Xiaoxu Guo, Yachao Cui

**Affiliations:** 1 School of Computer Science, Shaanxi Normal University, Xi’an, China; 2 School of Information and Intelligent Technology, Shaanxi Business College, Xi’an, China

**Keywords:** Tree-structured, Recursive neural network, Attention, *Child-Sum Tree-LSTMs*

## Abstract

**Background:**

Tree-structured neural networks have shown promise in extracting lexical representations of sentence syntactic structures, particularly in the detection of event triggers using recursive neural networks.

**Methods:**

In this study, we introduce an attention mechanism into *Child-Sum Tree-LSTMs* for the detection of biomedical event triggers. We incorporate previous researches on assigning attention weights to adjacent nodes and integrate this mechanism into *Child-Sum Tree-LSTMs* to improve the detection of event trigger words. We also address a limitation of shallow syntactic dependencies in *Child-Sum Tree-LSTMs* by integrating deep syntactic dependencies to enhance the effect of the attention mechanism.

**Results:**

Our proposed model, which integrates an enhanced attention mechanism into Tree-LSTM, shows the best performance for the MLEE and BioNLP’09 datasets. Moreover, our model outperforms almost all complex event categories for the BioNLP’09/11/13 test set.

**Conclusion:**

We evaluate the performance of our proposed model with the MLEE and BioNLP datasets and demonstrate the advantage of an enhanced attention mechanism in detecting biomedical event trigger words.

## Introduction

Biomedical event extraction technology can help researchers quickly and accurately locate events from a large number of biomedical literature, and represent them in a structured form. The task of biomedical event extraction addresses the deficiency of binary relations, defining more complex and fine-grained multisemantic relationships between entities. It has important research significance and application value in drug development, clinical assisted diagnosis and treatment, and the construction of biomedical ontology libraries. BioNLP’09 [1] proposed the biomedical event definition task for the first time. In the BioNLP’11 [2] shared task, all abstracts were retained based on the corpus of BioNLP’09, and some full texts were added. The GENIA corpus was used in the shared task to select nine event types from the ontology, which can be roughly divided into three categories from the perspective of element participation complexity. The dataset used in the BioNLP’13 [3] task comes not only from abstracts but also from entire corpora. The number of event types was adjusted to thirteen. Thus, the crucial and highly challenging task of biomedical event extraction has also received widespread attention. In biomedical event extraction, multisemantic relationships between fine-grained biological entities are extracted, which is of greatly significant for drug research and development, clinical diagnosis, and disease prevention.

**Fig. 1:**
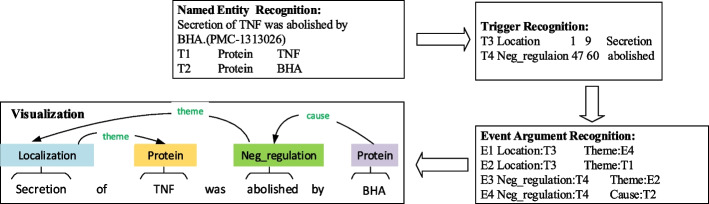
Biomedical event annotation and visualization. Entity name recognition is the first step. T1 and T2 denote two named entities “TNF/BHA”. The next step is to detect trigger words. T3 and T4 denote two trigger words “Secretion/abolished”, whose event types are *Location* and *Neg_regulaton*. The number 1 and 9 denote the trigger word *stimulates*’s start and end positions, respectively. Likewise, the number 47 and 60 denote the trigger word *abolished*’s start and end positions, respectively. E denotes the event type, which is decided by the trigger words. *Theme* and *Cause* refer to the event argument, which expresses the event’s semantic roles

The goal of biomedical event extraction is to identify the trigger words and related elements of a certain type of event. Trigger words refer to the words or phrases that trigger an event, whose type determines the type of event, while elements refer to the participants of the event and, can be biological entities or another event. The event arguments denote the participants in the event. The first stage extracts the trigger words, and the second extracts the arguments in the text. In the post-processing stage, it is necessary to combine the trigger words with the argument participating in the event to generate a complete event structure. Generally, biomedical event extraction employs a pipeline method, which divides the whole process into trigger word recognition, argument recognition and post-processing [4]. The graphical representation of a biomedical sentence with event annotation is illustrated in Fig. 1.

The types of biomedical events include simple events and complex events. The former have only one theme, while the latter have two types of elements, a theme and a cause. The argument of a flat event is a pair of elements composed of trigger words and entities, while the argument structure of nested events can be composed of trigger words and entities or only trigger words. The arguments of a nested event can be a trigger or an entity. Widely used models for event extraction include sequence models and recursive neural networks. LSTM, designed to solve the problem of vanishing and exploding gradients, is a special type of recurrent neural network (RNN). Additionally, LSTM [5,6] can maintain long-range dependencies and recognize the relationship between values at the beginning and end of a sequence. There are three gates in an LSTM model. The forget gate controls how much information the memory cell will receive from the memory cell from the previous step. The input gate controls how much information the current memory cell will receive from a potentially new memory cell. The output gate controls the value of the next hidden state. Tree-LSTM is designed to update memory cells that can memorize the sub-nodes’ history information recursively, and updates gating vectors and memory cells with the sub-nodes’ states [7-9].

Nevertheless, each sub-node has a different influence degree on the parent node. It will bring noise and affect the model training when all the hidden states of the child nodes are transferred to the parent nodes. We can focus on an important spot with an attention mechanism, and filter out unimportant information. Therefore, some studies have utilized attention mechanism to calculate the degree of dependencies between nodes [5]. In Tree-LSTM, each sub-node should be assigned a different weight. The existing shallow syntactic dependencies in *Child-Sum Tree-LSTMs* ignore deep syntactic dependencies. A deep syntactical structure refers to an abstract syntactic expression of a sentence, which can show the internal grammatical relationship and abstract syntactic structure between sentence components. We incorporate an attention mechanism into *Child-Sum Tree-LSTMs* to select more relevant nodes and collect sub-tree node states reasonably. To enhance the effect of attention mechanism, we integrate an enhanced attention mechanism into the *Child-Sum Tree-LSTMs* model using deep syntactic dependencies*.* The enhanced structure mines deep syntactic and semantic analysis of sentences. The evaluation results with the MLEE and BioNLP corpuses demonstrate the advantage of the enhanced attention mechanism. The primary contributions of this paper are listed as followings:

- We incorporate an attention mechanism into *Child-Sum Tree-LSTMs* for the task of biomedical event triggers.
- We update the node embedding in the predicate argument structure by the GAT method.
- We propose a novel model called *Child-Sum EATree-LSTM* to enhance the effect of the attention mechanism.

## Related works

Deep learning is to learn the internal laws and presentation of sample data. The information obtained in the learning process is very helpful to the interpretation of data such as text, images and sounds. Its ultimate goal is to enable machines to have the ability to analyze and learn like humans [10, 11]. Deep learning is a complex machine learning algorithm that has achieved results in speech and image recognition that far exceed previous related technologies.

In recent years, Tree-LSTM has been widely applied to many fields of NLP, such as text generation, neural machine translation, sentiment analysis, sentence semantic modelling, and event extraction tasks [12–15]. Compared with an sequence structure, a tree-structured neural network is a better alternative for extracting text information [16, 17]. In tree-structured neural network, words contribute unevenly to building a syntactic dependency tree, and it will bring noise and affect model training when all hidden states of the child nodes are transferred to the parent nodes. Therefore, each subnode should be allocated a different weight. Recently, some researchers have studied attention mechanism for tree-LSTM to more reasonably distribute attention weights. For example, Parikh et al. [18] utilized an attention mechanism to divide an object into sub-objects so that they could trivailly solve them in parallel on the SNLI dataset. Liu et al. [19] proposed attentive Tree-LSTM for sentence summarization. Chen et al. [20] generated sentence representations with a DCNN, and used attention pooling to obtain the most important information with Tree-LSTM in the pooling stage. Ahmed et al. [21] encoded a decomposable attention framework and the soft attention mechanism inside Tree-LSTM cell for semantic relatedness tasks. Liu et al. [22] proposed attentive tree-structured LSTM for VQA. To address the unbalanced distribution of weights, Shi et al. [23] proposed an attentive recursive neural network for sentence embedding, which integrated task-specific attention mechanism into Tree-LSTM. Geng et al. [24] utilized an attentive Tree-LSTM and sequential model respectively to extract semantic relation, and proved the effectiveness of the attention mechanism. To address the ignorance of existing methods in the complementary role and exploit the entire knowledge of the input sentence, Park et al. [25] proposed an attentive GCN to gather contextual and structural knowledge.

Inspired by previous works, we proposed a new attentive *Child-Sum Tree-LSTMs* for multi-classification task and developed the *Child_Sum EATree-LSTM* model, which considers the relationship between nodes more completely.

## Preliminaries

### Recursive neural network (RecurNN)

**Fig. 2:**
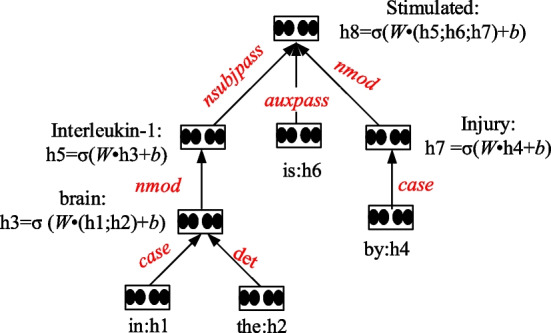
RecurNN encoding procedure

In a recurrent neural network (RNN) structure, a hidden layer’s input is the previous cell’s output. Sequential words are fed into an encoding block. An RNN can transfer and accumulate information in a timed sequence, and the probability of the last step could affect the next step. RecurNN deals with the length of variable inputs recursively. A tree-structured model is a good choice for the NLP task, because the syntactic structure tree can generate semantic relations. RecurNN is an artificial neural network (ANN) that has a tree-like hierarchical structure and the network nodes recurse the input information according to their connection order. Figure 2 illustrates the RecurNN encoding procedure.

In Fig. 2, h_i_ (i ∈ [1, N], N is the number of nodes) means the i-th node embedding. Each sub-node is connected to the parent node in pairs, and the sub-nodes and the parent node form a fully connected neural network.

The parent node embedding $h_{p}$ is computed as follows:

$$h_{p} = \sigma \left( {W \cdot \left( {X_{p\_1} ;\;X_{p\_2} ;\;X_{p\_i} ; \ldots ;X_{p\_n} } \right)} \right)$$

where n is the number of sub-node, $p\_i$ denotes the i-th child node of the parent node, and the operator; indicates the concatenation operation.

After encoding, the node vectors except for the leaf nodes are updated. Since the whole tree is formed recursively, each node is a representation of a subtree with its root. RecurNN aggregates the weight gradient and bias item of all layers.

### Predicate argument structure (PAS)

**Fig. 3:**
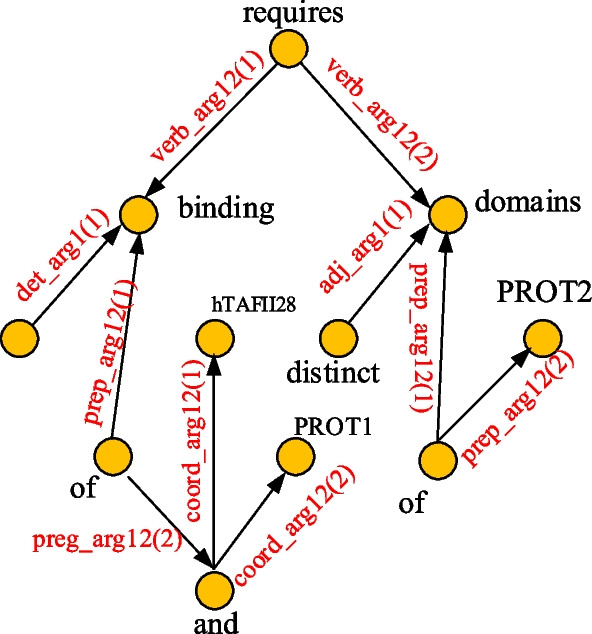
The predicate argument structure (PAS) of a sentence generated by the enju-genia parser

Enju-genia^1^ is trained with a biomedical corpus and is fit for the biomedical field. In this paper, an enju-genia parser analyser is used as a deep analyser, and it can output phrase structures and predicate-argument structures. The latter describes the relationship between words (phrases or clauses) in the form of graph. Predicate-argument relations look similar to labelled dependency structures. The parameter roles are divided into arg l-arg 4. Arg l indicates the subject of a verb or the target of a modifier. Arg 2 indicates the object of a verb or preposition. Arg N (N = 3 or 4) indicates the object or object complement of a verb, etc. A parameter usually refers to some words or phrases that can be used as subjects, verbs or prepositional objects. The parameters also include words or phrases that appear in a coordinate structure. An example of predicate argument structures is shown as Fig. 3.

The PAS can explore the deep syntactic and semantic relationships between words in sentences, and is composed of a predicate, and its corresponding parameter and role. Predicates are generally considered as verbs, sometimes they can also be other parts of speech, such as prepositions and conjunctions.

## Methodology

In the following, we first introduce the network architecture which is designed to detect biomedical event trigger words. Then we describe the core model of the network architecture in detail. Finally, we present the model training process.

### Network architecture

**Fig. 4:**
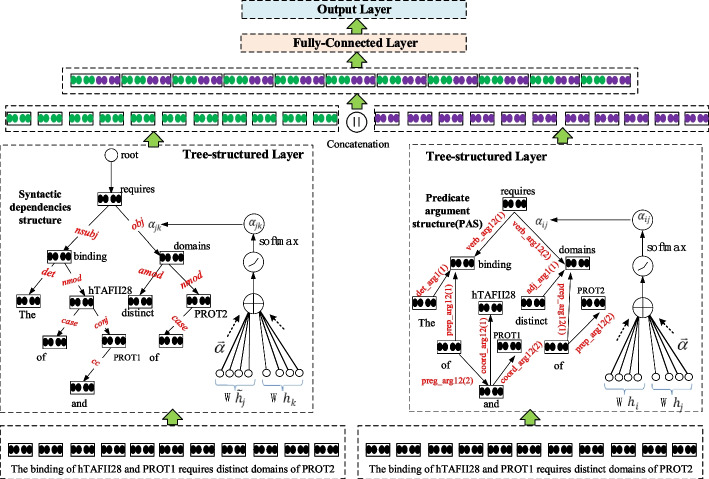
Network architecture

The proposed network architecture includes three layers, i.e., a tree-structured layer, a fully-connected layer and an output layer. Figure 4 describes the workflow of the components in the network architecture.

The overall network architecture to detect biomedical event trigger words. The syntactic dependencies structure is produced using the stanford toolkit.^2^${\widetilde{h}}_{j}$ is the cell state of the parent node j, and ${h}_{k}$ is the cell state of sub-node k. A PAS is produced by enju-genia. ${h}_{i}$ is the cell state of parent node i, and ${h}_{j}$ is the cell state of the neighbor node k. The weight coefficient ${a}_{ij}$ means the degree of syntactic dependencies between the node i and neighbor node j.

The network architecture employs the pre-training model SCIBERT [26] to initialize the word embedding. SCIBERT’s construction is nearly the same as that of BERT. SCIBERT constructs SCIVOCAB based on WordPiece [27], with a vocabulary of 30 K. Among the 1.14 M articles trained, 18% were in the computer field and 82% were in the biomedical field. Because a large part of the scientific corpus is about biomedical articles, the scientific vocabulary can also be regarded as biomedical vocabulary, which can evidently improve the performance of downstream biomedical tasks. In the tree-structured layer, we utilize the Stanford CoreNLP Natural Language Processing Toolkit [18] to produce CoNLL format dependencies, and the enju–genia parser to produce the predicate argument structure (PAS).The detailed principle of the *Child_Sum EATree-LSTM* model will be described in Sect. "Models". Word embedding is initialized by SCIBERT, and updated syntactically and semantically through the tree-structured layer. The fully-connected layer adopts the softmax function to classify trigger words, and the output layer will computes the probability value of the predicted results.

### Models

In the tree-structure layer, we adopt *Child-Sum Tree-LSTMs, Child-Sum ATree-LSTMs*, and *Child-Sum EATree-LSTMs* models to update the node embedding.

1. *Child-Sum Tree-LSTMs* [7, 8]

**Fig. 5:**
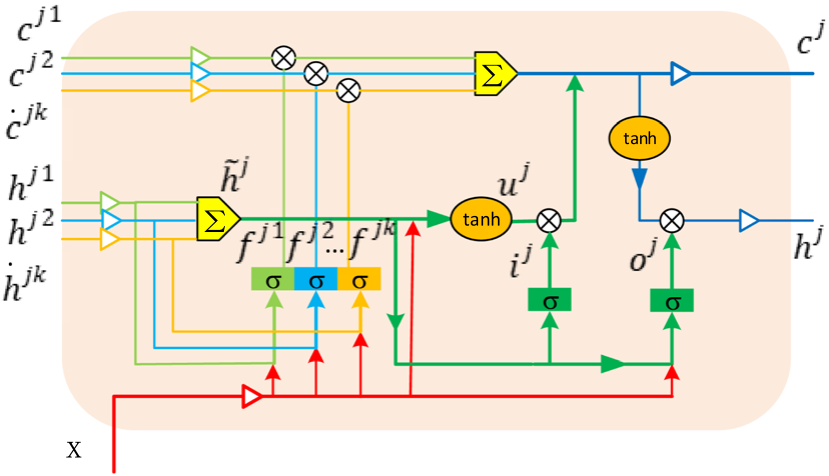
The detailed cell structure of *Child-Sum Tree-LSTMs*

The Tree-LSTM memory cell gathers the gated cell vectors of the sub-nodes. Therefore, they can reflect multiple descendant cells and capture the long-term dependecies over the structures. A sequential LSTM can be considered as a special case of Tree-LSTM [7]. *Child-Sum Tree-LSTMs* is suitable for trees with multiple branches or unordered child nodes, and it is constructed from a syntactic dependency structure, where the count of the dependencies for root can be highly variable. The cell structure of the *Child-Sum Tree-LSTMs* is illustrated in Fig. 5.

Suppose that $k \in C\left( j \right)$, where $C\left( j \right)$ denotes the j-th node’s subset. The input vector $x_{j}$ is the word embedding in a sentence*.* $c_{k}$ is the k-th cell state. $h_{k}$ is the k-th hidden state. ${ }i_{j}$ and $o_{j}$ are the input and output gates, respectively.${ }f_{k}$ is the k-th forgetting gate. The scope values of the three gates are all between 0 and 1. refers to the element-wise product operatoion, and ⊕ refers to the summation operation. *tanh* is a hyperbolic tangent function.

First, the parent node j aggregates the sub-nodes hidden state.

1 $$\tilde{h}_{j} = \mathop \sum \limits_{k \in C\left( j \right)} h_{k}$$

The forgetting gate is computed according to the input $x_{j}$ and the previous hidden state.

2 $$f_{jk} = \sigma \left( {W^{\left( f \right)} x_{j} + U^{\left( f \right)} h_{k} + b^{\left( f \right)} } \right)\quad k \in C\left( j \right)$$

where $W^{\left( f \right)}$, ${ }U^{\left( f \right)}$ and $b^{\left( f \right)}$ are the forgetting gate weight matrixes and bias item. σ denotes the activation function. Similarly, the input and output gates are updated in the same way.

3 $$i_{j} = \sigma \left( {W^{\left( i \right)} x_{j} + U^{\left( i \right)} \tilde{h}_{j} + b^{\left( i \right)} } \right)$$

4 $$o_{j} = \sigma \left( {W^{\left( o \right)} x_{j} + U^{\left( o \right)} \tilde{h}_{j} + b^{\left( o \right)} } \right)$$

where $W^{\left( i \right)}$, ${ }U^{\left( i \right)}$ and $b^{\left( i \right)}$ are the forgetting gate weight matrix and bias items, respsectively. $W^{\left( o \right)}$, ${ }U^{\left( o \right)}$ and $b^{\left( o \right)}$ are the output gate weight matrix and bias items, respectively.

5 $$u_{j} = \tanh \left( {W^{\left( u \right)} x_{j} + U^{\left( u \right)} \tilde{h}_{j} + b^{\left( u \right)} } \right)$$

In the forgetting gate, element-wise product operation is carried out with their corresponding cell state, and then summed with the element-wise product for input gate $i_{j}$ and $u_{j}$.

6 $$c_{j} = i_{j} \odot u_{j} + \sum\limits_{k \in C\left( j \right)} {f_{jk} } \odot c_{k}$$

7 $$h_{j} = o_{j} \odot \tanh c_{j}$$

where operation $\odot$ refers to element-wise product operation.

Then, we will compute the j-th cell state and hidden state.

(2) Attentive *Child-Sum Tree-LSTMs *(*Child-Sum ATree-LSTMs*)

*Child-Sum Tree-LSTMs* combine information from multiple sub units, and sum it over the subhidden states of all child nodes. However, the contribution degree of the child nodes to the parent node is different. It will bring noise and affect the model training when all the hidden layer states of the child nodes are transferred to the parent nodes. The attention mechanism focuses on the more relevant nodes, and distributes the importance of each sub-tree component to reasonably construct the whole tree [5]. We summarize the purpose of the attention mechanism in *Child-Sum Tree-LSTMs* as follows: (1) Assign different weights to each node; (2) Pay more attention to important nodes and ignore unimportant ones; and (3) Pay attention to the global information at the same time while processing local information.

**Fig. 6:**
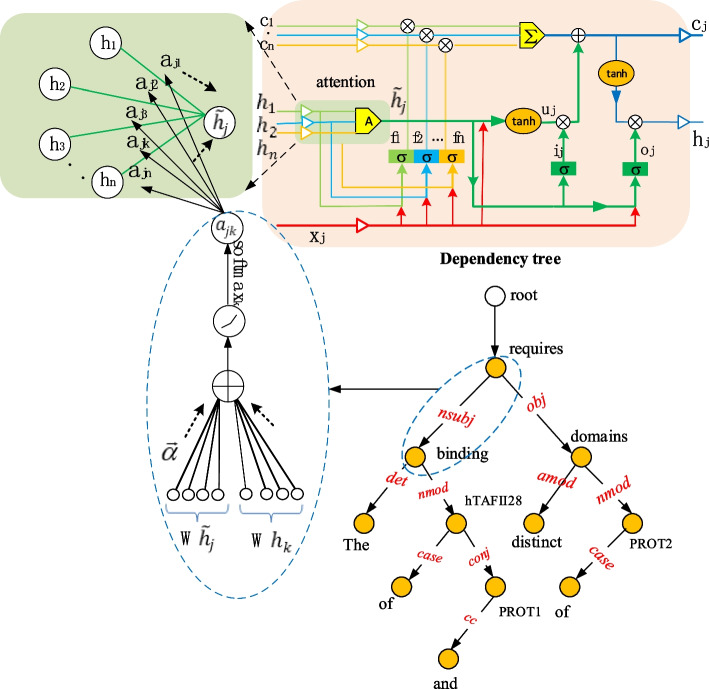
The structure of *Child-Sum ATree-LSTMs*

Therefore, we incorporate an attention mechanism into *Child-Sum Tree-LSTMs*, refered to as *Child-Sum ATree-LSTMs*. The way that *Child-Sum ATree-LSTMs* converge the hidden state is by adopting the weighted sum method, replacing the method of summing the sub node state directly in the original model. The structure of *Child-Sum ATree-LSTMs* is illustrated in Fig. 6.

In Fig. 6, ‘A’ indicates the attention mechanism. The child nodes’ hidden states in the tree are converged to the parent node with the attention mechanism. RecurNN is used with the CoNLL-07 dependency tree. We take a branch (*requires*- > *binding*) in the tree structure as an example. The branch to the left blue ellipse is enlarged. $\tilde{h}_{j}$ is the node *requires* vector, and $h_{k}$ is the node *binding* vector. $\vec{a}$ is the weight vector.  indicates the activation function.

The weighted summation coefficient is calculated as follows:

8 $$a_{jk} = \frac{{{\text{exp}}(LeakyReLU(\vec{a}^{T} [W\tilde{h}_{j} ||Wh_{k} ])}}{{\mathop \sum \nolimits_{k \in child\left( j \right)} {\text{exp}}(LeakyReLU(\vec{a}^{T} [W\tilde{h}_{j} ||Wh_{k} ])}}$$

LeakyReLU is a nonlinear activation function [28].

The weighted vector $\tilde{h}_{j}$ is produced by the summation of each node assigned respective attention coefficients ${ }a_{jk}$.

9 $$\tilde{h}_{j} = \mathop \sum \limits_{k \in child\left( j \right)} a_{jk} h_{k}$$

10 $$f_{jk} = \sigma \left( {W^{\left( f \right)} x_{j} + U^{\left( f \right)} h_{k} + b^{\left( f \right)} } \right)\quad k \in C\left( j \right)$$

11 $$i_{j} = \sigma \left( {W^{\left( i \right)} x_{j} + U^{\left( i \right)} \tilde{h}_{j} + b^{\left( i \right)} } \right)$$

12 $$o_{j} = \sigma \left( {W^{\left( o \right)} x_{j} + U^{\left( o \right)} \tilde{h}_{j} + b^{\left( o \right)} } \right)$$

13 $$u_{j} = \tanh \left( {W^{\left( u \right)} x_{j} + U^{\left( u \right)} \tilde{h}_{j} + b^{\left( u \right)} } \right)$$

14 $$c_{j} = i_{j} \odot u_{j} + \mathop \sum \limits_{k \in C\left( j \right)} f_{jk} \odot c_{k}$$

15 $$h_{j} = o_{j} \odot \tanh c_{j}$$

(3) Enhanced *Child-Sum ATree-LSTMs *(*Child-Sum EATree-LSTMs*)

The deep parser constructs a graph structure to show the internal grammatical relationships and abstract syntactic structure between sentence components. Such grammatical relationships and syntactic structures cannot be shown with a shallow dependency. Unlike the dependency parser, which analyzes the superficial relationship between words, the deep parser focuses more on finding the deep syntactic and semantic relationship between words.

**Fig. 7:**
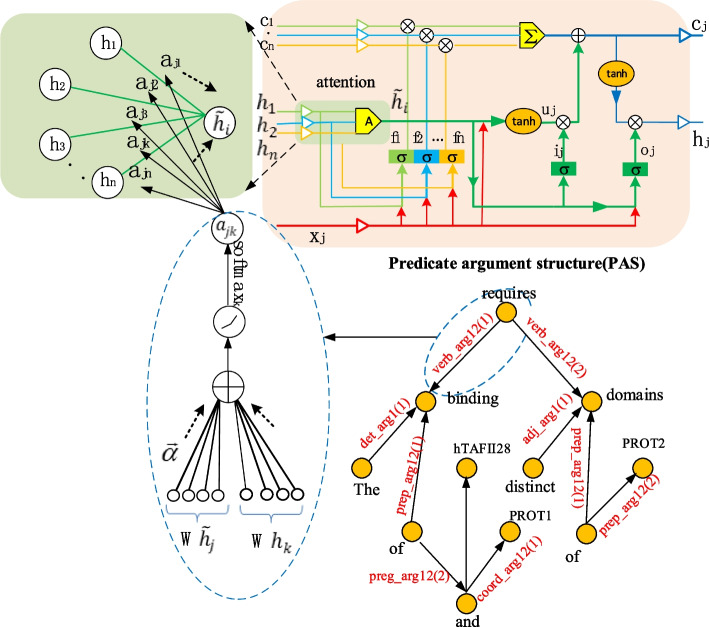
The structure of *Child-Sum EATree-LSTMs*

In this paper, we not only construct a CoNLL-2007 dependency tree with shallow parsing, but also construct PAS with deep parsing. PAS cannot be used for RecurNN because it is not a tree structure. However, we can update node vector of PAS by the methods such as GAT [22]. Inspired by this idea, we attempt to the enhance attention effect of *Child-Sum ATree-LSTMs* with deep parsing. The enhanced structure considers the relationships between nodes completely. We develop *Child-Sum EATree-LSTM*, which integrates an enhanced attention mechanism into the *Child-Sum Tree-LSTMs* model*.* The structure of *Child-Sum EATree-LSTM*s is illustrated in Fig. 7.

In the PAS, the edge between *requires* and *binding* is enlarged to the left blue ellipse. $h_{i}$ is the node *requires vector,* and $h_{j}$ is the node *binding* vector. We compute the weighted summation coefficient in the PAS [28].

16 $$a_{jk} = \frac{{{\text{exp}}(LeakyReLU(\vec{a}^{T} [W\tilde{h}_{j} ||Wh_{k} ])}}{{\mathop \sum \nolimits_{{k \in {\mathcal{N}}_{j} }} {\text{exp}}(LeakyReLU(\vec{a}^{T} [W\tilde{h}_{j} ||Wh_{k} ])}}$$

The weight coefficient $a_{jk}$ denotes the dependency degree between node j and neighbor node k.

The weight coefficient $\rho$ is learned during the model training process.

The weighted vector $\tilde{h}_{j}$ is produced by the summation of each node assigned respective attention coefficients:

17 $$\tilde{h}_{j} = \mathop \sum \limits_{k \in C\left( j \right)} a_{jk} h_{j}$$

$a_{jk}$ is the attention weight coefficient on each subhidden state in a subtree.

18 $$f_{jk} = \sigma \left( {W^{\left( f \right)} x_{j} + U^{\left( f \right)} h_{k} + b^{\left( f \right)} } \right)\quad k \in C\left( j \right)$$

19 $$i_{j} = \sigma \left( {W^{\left( i \right)} x_{j} + U^{\left( i \right)} \tilde{h}_{j} + b^{\left( i \right)} } \right)$$

20 $$o_{j} = \sigma \left( {W^{\left( o \right)} x_{j} + U^{\left( o \right)} \tilde{h}_{j} + b^{\left( o \right)} } \right)$$

21 $$u_{j} = \tanh \left( {W^{\left( u \right)} x_{j} + U^{\left( u \right)} \tilde{h}_{j} + b^{\left( u \right)} } \right)$$

22 $$c_{j} = i_{j} \odot u_{j} + \mathop \sum \limits_{k \in C\left( j \right)} f_{jk} \odot c_{k}$$

23 $$h_{j} = o_{j} \odot \tanh c_{j}$$

Next, we will concatenate the hidden state of PAS computed by Formula (23) with that of dependency parsing tree computed by Formula (15). Then, we feed the results into the fully-connected layer, and the algorithm runs as in Fig. 4.

### Model training

The training process of RecurNN is similar to that of an RNN. The difference between them is that the former back-propagates the residual from top to bottom, while the latter back-propagates the residual from output to input. The overall training process of the model is illustrated in Algorithm 1. 

The residual error is back-propagated from the parent to child nodes over the structures. The objective function minimizes the overall cross-entropy errors. We set* E* as the loss function, and the RecurNN training uses the gradient descend method to minimize objective function $J\left( \theta \right)$.

24 $$J\left( \theta \right) = - \frac{1}{k}\mathop \sum \limits_{i}^{k} log(\hat{p}_{{}} \left( {yE_{j} \left( x \right)} \right)) + \frac{{}}{2}\theta_{2}^{2}$$

where θ denotes the parameter set*. k* is the number of labelled nodes. $\uplambda$ denotes an L2 regulation hyperparameter.

25 $$\frac{\partial E}{{\partial W}} = \mathop \sum \limits_{l} \frac{\partial E}{{\partial W^{l} }}, \frac{ \partial E}{{\partial b}} = \mathop \sum \limits_{l} \frac{\partial E}{{\partial b^{l} }}$$

where l is the layers of the network.

The final weight gradient and bias of each layer were accumlated. The backprogatoin algorithm propagates errors from the output to the input. The algorithm updates weight as follows:

26 $$W \leftarrow W + \frac{\partial E}{{\partial W}},{ }b \leftarrow b + \frac{\partial E}{{\partial b}}$$

where $\upeta$ denotes the learning rate.

## Results and discussion

In this section, we first introduce the experimental metrics, datasets and hyper-parameters settings. Next, we perform experiments to verify the effect of the attention mechanism and hybrid attention mechanism. After confirming the advantage of the hybrid model, we compare it with baselines. Finally, we discuss the experimental result and analyze the errors.

### Experimental metric

Precision refers to the proportion of positive samples in the positive cases determined by the classifier, and recall refers to the proportion of predicted positive cases in the total positive cases. The F1-score is a measure of classification, and the value ranges from 0 to 1. For multi-classification task, the F1-score is often used as an evaluation metric, which is the harmonic average of the accuracy rate and recall rate. The F1-score is computed as follows:

$$F_{1} = 2 \times \frac{precsion \times recall}{{precision + recall}}$$

In our experiment, we use the F1-score as the evaluation metric.

### Dataset and hyper-parameters settings

#### Datasets

In this paper, we performed experiment with the MLEE** a**nd BioNLP datasets. MLEE contains enriched levels of biomedical events. Table 1 shows the statistics of the MLEE and BioNLP’09 datasets. The MLEE dataset includes 262 samples containing 19 types of biomedical events across levels of biological organization from the molecular level to the organ system. All events can be divided into 19 sub-classes, and further disassembled into 19 subcategories.

**Table 1:** Statistics of datasets

Dataset	Documents	Sentences		Event
*MLEE*				
Train	206	1825	4673	
Validation	30	260	668	
Test	59	523	1336	
Total	295	2608	6677	
*BioNLP’09*				
Train	800	7449	8597	
Validation	150	1450	1809	
Test	260	2447	3182	
Total	1210	11,346	13,588	

#### Computational environment and setup

Table 2 lists the hyperparameters and computational environments in our experiments.

**Table 2:** Hyper-parameters and computational environments

Hyper-parameters	Computational environments		
parameters	value	parameters	value
Batch size	32	CPU	Inter (R) Xeon (R) E5-268W
Dropout	0.2	GPU	GTX 1080Ti
Learning rate	0.001	Memory	128G
Epochs	50	parser	Python 3.6
Optimizer	Adam	tool	Pytorch 1.9
Word embedding	720	IDE	Pycharm Professional 2022
Hidden units	256		
L2 regularization	0.003		

The Adam optimizer combines the advantages of AdaGrad and RMSProp optimization algorithms. The first moment estimation (i.e. the mean of the gradient) and the second moment estimation (i.e. the non centralized variance of the gradient) of the gradient are comprehensively considered to calculate the update step. We apply Adam optimizer for the optimal algorithm performance, with the 1st momentum coefficient β_1_ = 0.88 and the 2nd momentum coefficientβ_2_ = 0.90.

### Experimental results

#### Ablation experiment

To verify the effect of the hybrid attention mechanism, we conduct an ablation experiment to record the changes in the weight coefficient for four situations and draw the corresponding heatmaps.

**Case**: *The binding of hTAFII28 and PROT1 requires distinct domains of PROT2.*

**Fig. 8:**
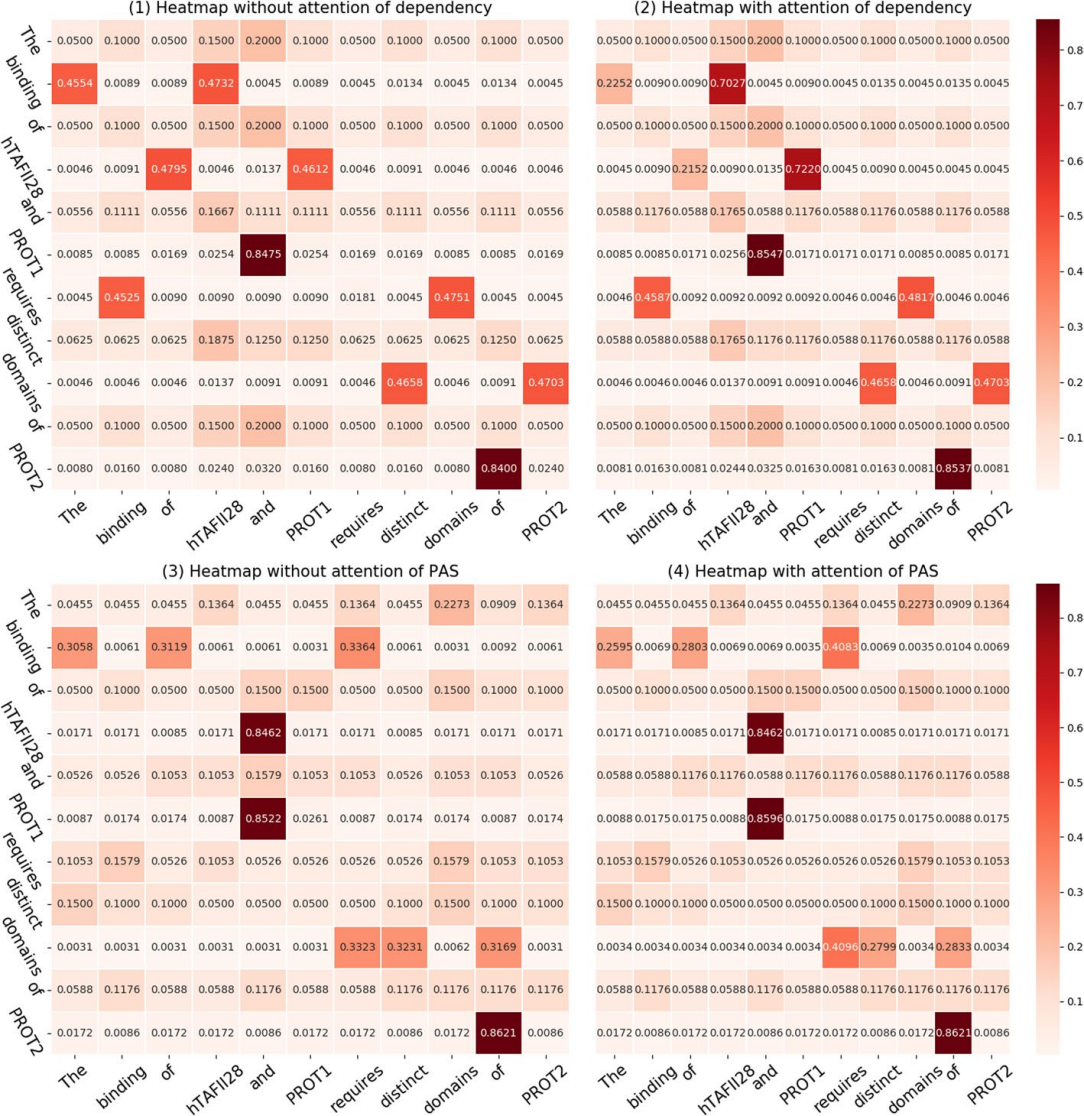
Comparison heatmaps of the four attention weight coefficients. The scales of the abscissa and ordinate represent the words in the sentence. The words on the ordinate depend on the words on the abscissa. The numbers denote the correlation coefficients. The colour depth represents the degree of dependency between words. The darker the colour is, the stronger the dependencies between words, while the lighter the colour is, the weaker the dependencies between words

The visual results of attentional weight coefficient changes are shown in Fig. 8:

From Fig. 8(1), we can see that the degree of dependency between words is relatively uniform. After integrating the attention mechanism, the values appear to changed. For example, the subnodes of node *binding* include *The* and *hTAFII28*. The correlation coefficient is 0.4554 between *binding* and *The* and 0.4732 between *binding* and *hTAFII28.* After incorporating attention into the syntactic dependency, Fig. 8(2) shows that the values have obviously changed between the parent node *binding* and the two subnodes *The* and *hTAFII28*. The reason is that the dependency degrees between the parent and two subnodes are different. Figure 8(3) shows that the degree of dependency between words is relatively uniform in the PAS. However, neighbour nodes have different dependencies on the central node. Therefore, the correlation coefficient score is changed after incorporating the attention mechanism into the PAS. For example, the neighbour nodes of the node *domains* include *requires*, *distinct*, and *of*. The correlation coefficients are 0.3323, 0.3231, and 0.3169, respectively. After integrating the attention mechanism, the values are changed to 0.4096, 0.2799, and 0.2833, respectively. The reason is that the dependency degrees between the node and three neighbour nodes are different.

In addition, we compare *Child-Sum EATree-LSTMs* with those that miss some model components.

**Fig. 9:**
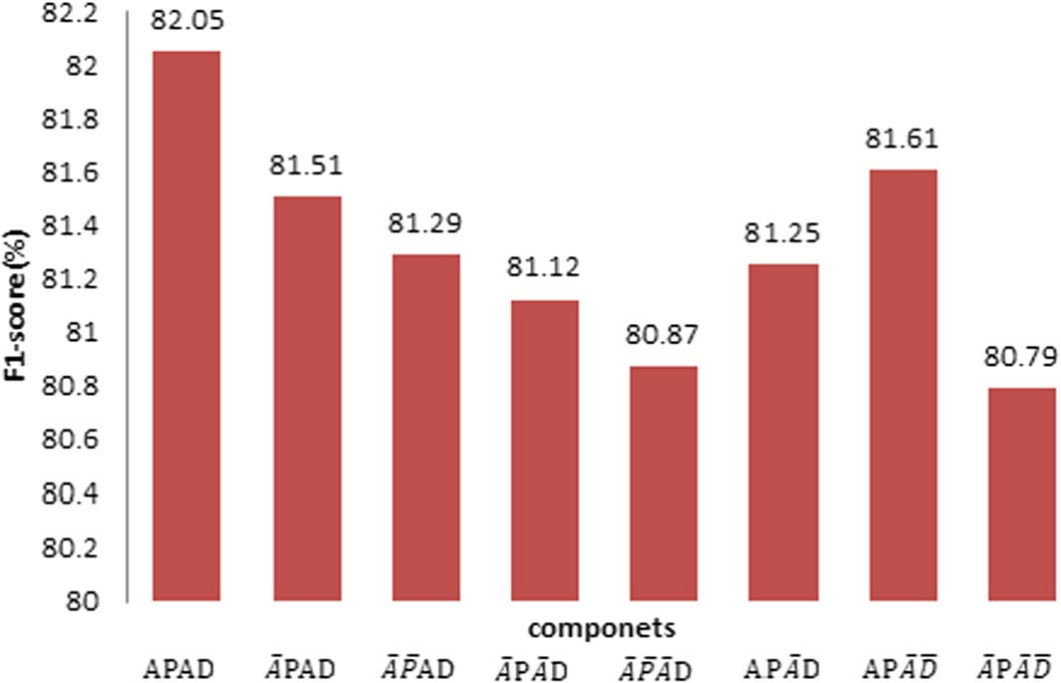
Ablation experiment histograms with the MLEE dataset

Table 3 describes the missing component. The ablation experiment results shown in Fig. 9 verify the effectiveness of the proposed components. We can see from Fig. 9 that the replacement of any component will reduce the model performance. After reducing the attention mechanism in the PAS for *Child_Sum EATree-LSTMs*, the F1-score is reduced by 0.54% (APAD vs. $\overline{A}\mathrm{PAD }$). In the same way, if we reduce the attention mechanism in the PAS and the dependency, the F1-score will be reduced by 0.13% ($\overline{A}\mathrm{P }\overline{A}\mathrm{D }$ vs. $\mathrm{AP}\overline{A}\mathrm{D }$). The F1-score is increased by 0.82% when the attention mechanism is incorporated into the PAS. The effectiveness of the attention mechanism is proven.

**Table 3:** Component descriptions

Component	Description
$\mathrm{APAD}$	*Child_Sum EATree-LSTMs*
$\overline{A}\mathrm{PAD }$	Reduce attention in the PAS
$\overline{A }\overline{P}\mathrm{AD }$	Reduce the PAS
$\overline{A}\mathrm{P }\overline{A}\mathrm{D }$	Reduce attention in the PAS and the dependency
$\overline{A }\overline{P }\overline{A}\mathrm{D }$	Retain the dependency only
$\mathrm{AP}\overline{A}\mathrm{D }$	Reduce attention in the dependency
$\mathrm{AP}\overline{A }\overline{D }$	Reduce the dependency
$\overline{A}\mathrm{P }\overline{A }\overline{D }$	Reduce attention in the PAS

When we reduce the PAS in *Child-Sum EATree-LSTMs*, the F1-score is reduced by 0.22% ($\overline{A}\mathrm{PAD }$ vs. $\overline{\mathrm{A} }\overline{\mathrm{P}}\mathrm{AD }$). If we replace dependency with PAS, the F1-score will be increased by 0.32%. The results demonstrate that integrating deep syntactic dependencies can enhance the ability to learn the tree representation.

#### Experimental results and analysis

To prove the advantage of the hybrid attention mechanism, we compare with baselines as follows:

- The RecurCRF [29] model was applied to RecurNN to vectorize the whole dependency tree to extract dependency-based syntax information completely.
- He et al. [30] proposed a fine-grained method with multilevel attention and sentence embeddings.
- Wang et al. [31] presented an end-to-end model that uses the probability distribution of triggers and the syntactic structure in an attention-based gate GCN.
- Ahmed et al. [21] encoded decomposable attention framework and the soft attention mechanism inside a Tree-LSTM cell on semantic relatedness tasks.
- Nie et al. [32] proposed a word EANNP model for event extraction task to construct semantic information between words and obtain the words’ meaning.
- Riedel et al. [33] proposed a joint model to extract biomedical event on the four BioNLP'09/'11 shared tasks.
- Björne et al. [34,35] developed a model to extract complex events among proteins and genes in biomedical text, and the model performance achieved a high score on three subtasks.
- Yu et al. [36] proposed an end-to-end model with Bi-LSTM and Tree-LSTM for extracting event task. Bi-LSTM is used to learn the semantic and syntactic information between sentences, and Tree-LSTM is employed to recognize the relationships between target pairs.
- Hakala et al. [37] applied EVEX system, which is a text mining tool with events extracted from PubMed Central and PubMed abstracts for BioNLP Shared Tasks.
- To avoid cascading errors and semantic missing, Li et al. [38] employed rich features and dual decomposition to integrate word vectors to extract events.
- Zhou et al. [39] presented a novel model to detect event triggers with domain knowledge.

After confirming the advantage of the enhanced model, we compare it with other models, and the results are presented in Table 4.

**Table 4:** Performance of different models

Dataset	Method	Precision (%)	Recall (%)	F1-score (%)
MLEE	RecurCRF [29]	81.12	79.15	80.28
	Yan Wang [31]	82.20	78.25	80.18
	Xinyu He [30]	82.01	78.02	79.96
	Attentive Child_Sum Tree-LSTM	82.95 (82.75 ± 0.19)	80.62 (80.41 ± 0.21)	81.77 (81.51 $\pm \hspace{0.17em}$0.19)
	*Child_Sum EATree-LSTM*	83.24 (83.00 ± 0.19)	80.90 (80.71 ± 0.21)	**82.05 (81.96 **$\pm \hspace{0.17em}$**0.19)**
BioNLP’09	RecurCRF	76.42	70.45	73.24
	Attentive Child_Sum Tree-LSTM	75.95 (75.71 ± 0.19)	72.23 (72.01 ± 0.21)	74.11 (73.90 $\pm \hspace{0.17em}$0.19)
	*Child_Sum EATree-LSTM*	76.84 (76.64 ± 0.19)	73.35 (73.11 ± 0.21)	**75.05 (74.86 **$\pm \hspace{0.17em}$**0.19)**

The best results are marked in bold

Table 4 shows that the Tree-LSTM model with an attention mechanism performs better than *RecurCRF,* which has no attention mechanism. The proposed *Child_Sum EATree-LSTM* model scores competitively against baselines with the MLEE and BioNLP’09 corpus. The best performance is obtained using our proposed model, which integrates an enhanced attention mechanism into Tree-LSTM, with MLEE and BioNLP’09.

The events triggers on the MLEE dataset are classified into 4 categories including *Molecular*, *Anatomical*, *Planned*, *General*, and decomposed into 19 subcategories. The event triggers on the MLEE dataset are broadly divided into 3 categories including *SVT*, *BIND*, and *REG*, and can be further decomposed into 9 subcategories. We compared all the complex subcategories with those of the other models in detail. Table 5 lists the detailed complex event trigger detection results on MLEE dataset.

**Table 5:** Detailed complex event trigger detection results on MLEE dataset

Method	Pyysalo et al. [4]	Zhou et al. [39]	Nie et al. [32]	*Child_Sum EATree-LSTM*
Event Type	P/R/F1 (%)	P/R/F1 (%)	P/R/F1 (%)	P/R/F1 (%)
Binding	84.00/76.36/80.00	81.13/78.18/79.63	81.82/80.36/81.08	82.02/80.55/**81.28**
Regulation	46.48/60.37/52.52	56.49/53.05/54.72	59.90/67.98/63.68	61.25/69.21/**64.99**
Positive Regulation	67.85/86.73/76.14	71.58/86.41/78.30	67.14/91.03/77.28	72.02/87.25/**78.91**
Negative Regulation	74.35/77.03/75.66	77.09/78.83/77.95	70.86/84.55/77.10	75.12/81.22/**78.05**

The best results are marked in bold

**Fig. 10:**
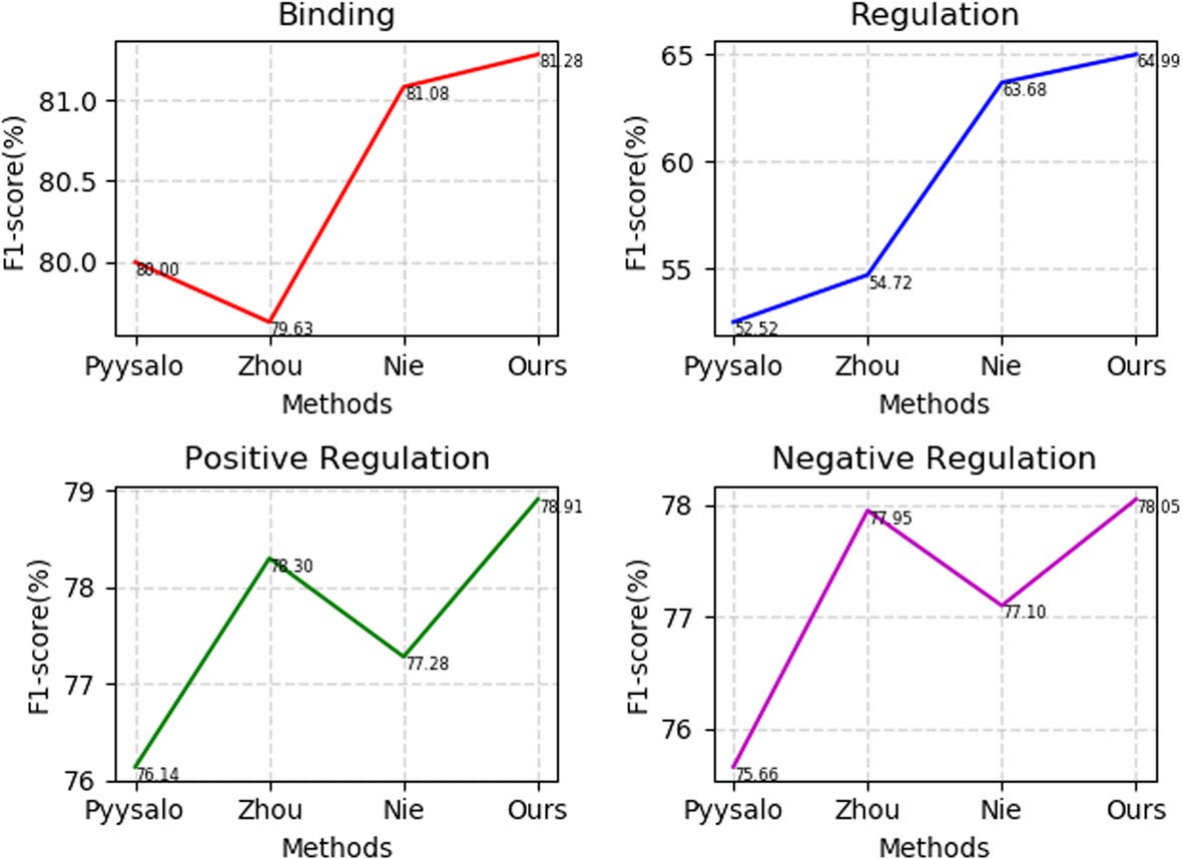
Line charts of the results in Table 5

The *general* category type on MLEE dataset takes multiple arguments and may refer to nested or overlapping arguments. *Binding* event has at least one *theme*, and *regulation* event indicates regulatory events and causal relationship, which has two types of roles (*theme* or *cause*). The *regulation* event maybe contain another nested event. It can be observed from Fig. 10 that *Child-Sum EATree-LSTM* outperforms the baselines.

To further prove whether the hybrid attention strategy is effective for a nested event on other datasets or not, we compare it with other models with the BioNLP’09/’11/13 test set for the complex event subcategories. The detailed results on the different dataset is shown in Table 6.

**Table 6:** Detailed nested event trigger detection results on the different datasets

Dataset	Method	Event Type	
		BIND P/R/F1 (%)	REG P/R/F1 (%)
BioNLP’09	Riedel et al. [33]	–/–/52.6	58.33/48.76/53.12
	J Björne et al. [34]	–/–/–	–/–46.9
	Yu et al. [36]	65.47/44.69/53.12	–/–/–
	*Child-Sum EATree-LSTM*	66.12/45.12/**53.64**	58.55/49.12/**53.42**
BioNLP’11	J Björne et al. [35]	43.60/42.97/43.28	47.64/38.72/42.72
	Riedel et al. [33]	56.42/42.97/48.79	52.67/37.52/43.82
	Yu et al. [36]	60.23/47.10/52.86	42.42/48.76/45.37
	*Child-Sum EATree-LSTM*	61.12/47.25/**53.30**	53.25/41.25/**46.49**
BioNLP’13	Hakala et al. [37]	–/–/42.88	–/–/38.41
	Li et al. [38]	45.43/46.25/45.83	47.81/34.21/39.88
	Yu et al. [36]	45.76/47.28/46.51	46.31/38.97/42.32
	*Child-Sum EATree-LSTM*	47.88/47.32/**47.60**	47.99/38.25/**42.57**

The best results are marked in bold

BIND denotes *Binding*. REG denotes *Regulation*, *Positive Regulation*, and *Negative Regulation*

**Fig. 11:**
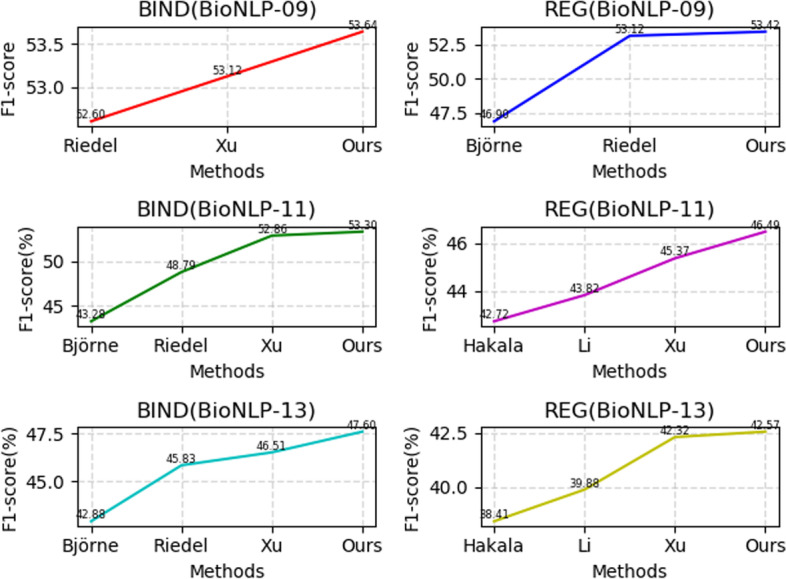
Line charts of the results in Table 6

It is observed from Table 6 and Fig. 11 that we can reach the conclusion that is consistent with that from previous experimental results. The proposed *Child-Sum EATree-LSTM* model achieves better performance on almost all of the complex event categories for the BioNLP’09/11/13 test sets. The enhanced attentive Tree-LSTM can deeply and completely mine nested and overlapping events, which consider the argument information. The reason is that (1) complex events are iterative in nature. Therefore, we choose a recursive neural network to detect complex event triggers, and (2) integrating the attention mechanism into Tree-LSTM to focus on the information that is more critical to the current task among the multiple sub node inputs can effectively extract nested or overlapping events.

#### Error analysis

The first reason leading to event extraction errors is that the missing and undetected entities by tools can influence the following event extraction operations. For the second error, we consider that it is due to the scarcity and imbalance of training sets, which may be alleviated by transfer learning. In addition, the number of trigger words is the main cause leading to errors. A trigger word is not only a single word. According to statistics, approximately 8% of trigger words are composed of multiple words [35]. These words appear to be in the corpus diversely. For example, *role* can serve as a trigger word in the training data. However, the phase with a degree modification before it can also serve as a trigger word, such as *critical role* and *potential role*. The above trigger candidates may not always appear as trigger words, and their appearance may also represent words that do not participate in the event.

## Conclusion and future work

We incorporate the attention mechanism into *Child-Sum Tree-LSTMs* to select more relevant nodes and collect sub-tree node state reasonably. Then we update the node embedding in PAS by the method of GAT. And lastly, we incorporate the enhanced attention mechanism into *Child-Sum Tree-LSTM*. The model not only filters out redundant information in the syntax dependency tree, but also deep the internal grammatical relationship and abstract syntactic structure between sentence components. The proposed *Child-Sum EATree-LSTM* model achieves the better performance on almost all of the complex event categories. Our results demonstrate the effectiveness of the enhanced attention mechanism. In future work, we will explore new vector concatenating methods, and conduct more thorough experiments.

## Data Availability

All data generated or analysed during this study are included in this published article. The data have been deposited in the website. Requests for material should be made to the corresponding authors. http://nactem.ac.uk/MLEE/, https://2011.bionlp-st.org/bionlp-shared-task-2011, https://2013.bionlp-st.org/introduction.

## Footnotes

1 A predicate argument structure(PAS) is produced by the enju-genia parser. (https://mynlp.is.s.u-tokyo.ac.jp/enju/examples.html).

2 Dependency tree is produced by the Stanford CoreNLP Natural Language Processing Toolkit^[[[[[20]]]]]^ (https://stanfordnlp.github.io/CoreNLP/).
